# Branched-Chain Amino Acid Ingestion Stimulates Muscle Myofibrillar Protein Synthesis following Resistance Exercise in Humans

**DOI:** 10.3389/fphys.2017.00390

**Published:** 2017-06-07

**Authors:** Sarah R. Jackman, Oliver C. Witard, Andrew Philp, Gareth A. Wallis, Keith Baar, Kevin D. Tipton

**Affiliations:** ^1^Sport and Health Sciences, College of Life and Environmental Sciences, University of ExeterExeter, United Kingdom; ^2^Physiology, Exercise and Nutrition Research Group, University of StirlingStirling, United Kingdom; ^3^School of Sport, Exercise and Rehabilitation Sciences, University of BirminghamBirmingham, United Kingdom; ^4^Department of Neurobiology, Physiology and Behavior, University of California, DavisDavis, CA, United States

**Keywords:** amino acid ingestion, fractional synthesis rate, intracellular signaling proteins, leucine, muscle anabolism

## Abstract

The ingestion of intact protein or essential amino acids (EAA) stimulates mechanistic target of rapamycin complex-1 (mTORC1) signaling and muscle protein synthesis (MPS) following resistance exercise. The purpose of this study was to investigate the response of myofibrillar-MPS to ingestion of branched-chain amino acids (BCAAs) only (i.e., without concurrent ingestion of other EAA, intact protein, or other macronutrients) following resistance exercise in humans. Ten young (20.1 ± 1.3 years), resistance-trained men completed two trials, ingesting either 5.6 g BCAA or a placebo (PLA) drink immediately after resistance exercise. Myofibrillar-MPS was measured during exercise recovery with a primed, constant infusion of L-[ring^13^C_6_] phenylalanine and collection of muscle biopsies pre and 4 h-post drink ingestion. Blood samples were collected at time-points before and after drink ingestion. Western blotting was used to measure the phosphorylation status of mTORC1 signaling proteins in biopsies collected pre, 1-, and 4 h-post drink. The percentage increase from baseline in plasma leucine (300 ± 96%), isoleucine (300 ± 88%), and valine (144 ± 59%) concentrations peaked 0.5 h-post drink in BCAA. A greater phosphorylation status of S6K1^Thr389^ (*P* = 0.017) and PRAS40 (*P* = 0.037) was observed in BCAA than PLA at 1 h-post drink ingestion. Myofibrillar-MPS was 22% higher (*P* = 0.012) in BCAA (0.110 ± 0.009%/h) than PLA (0.090 ± 0.006%/h). Phenylalanine Ra was ~6% lower in BCAA (18.00 ± 4.31 μmol·kgBM^−1^) than PLA (21.75 ± 4.89 μmol·kgBM^−1^; *P* = 0.028) after drink ingestion. We conclude that ingesting BCAAs alone increases the post-exercise stimulation of myofibrillar-MPS and phosphorylation status mTORC1 signaling.

## Introduction

It is well-established that ingestion of essential amino acids (EAA) following resistance exercise stimulates an increased response of muscle protein synthesis MPS in humans (Smith et al., 1998; Tipton et al., 1999a). Indeed, the stimulation of MPS in humans can be achieved by supplying EAA only (i.e., the non-EAAs necessary for MPS may be supplied by endogenous sources) (Tipton et al., 1999b). More recent evidence from studies in rodents and cell culture models suggest that the stimulation of MPS by EAA may be mediated by a few amino acids rather than a combination of all EAA (Crozier et al., 2005; Kimball and Jefferson, 2006). The branched-chain amino acid (BCAA), leucine, has been shown to play a unique role in stimulating MPS (Kimball and Jefferson, 2006). Leucine serves as substrate for the synthesis of new muscle proteins and as a signal to initiate the rate-limiting translation initiation step of MPS (Crozier et al., 2005). Accordingly, the response of MPS to leucine provision has been extensively investigated over the past two decades, both in cell culture studies (Buse and Reid, 1975; Kimball and Jefferson, 2006; Atherton et al., 2010b) and *in vivo* rodent (Anthony et al., 1999, 2000; Crozier et al., 2005; Norton et al., 2012) and human (Matthews, 2005; Wilkinson et al., 2013) studies.

The stimulation of MPS is accompanied by an increased activation of intracellular signaling proteins that regulate the translational activity of MPS (Philp et al., 2011). In particular, the mechanistic/mammalian target of rapamycin complex-1 (mTORC1) signaling, often assessed as the phosphorylation status of the 70 kDa ribosomal S6 protein kinase (S6K1), is stimulated by ingestion of EAA following resistance exercise (Dreyer et al., 2008; Moberg et al., 2016). There is current debate over whether leucine alone (Churchward-Venne et al., 2014), or the BCAAs combined (Moberg et al., 2016), provide the most important component of an exogenous EAA source for stimulating the mTORC1-S6K1 signaling pathway. Whereas, the inclusion of leucine is necessary for the maximal activation of mTORC1 signaling (Moberg et al., 2014), recent results from the same research group show that mTORC1 signaling is enhanced with the addition of the other two BCAAs, valine and isoleucine (Moberg et al., 2016). Moreover, there often is a disconnect between the response of mTORC1 signaling and MPS (Witard et al., 2009; Atherton et al., 2010a; Glynn et al., 2010; Macnaughton et al., 2016; McGlory et al., 2016). Thus, the response of MPS to BCAA ingestion is still uncertain.

In humans, the ingestion of leucine alone has been shown to stimulate an increased response of MPS at rest (Wilkinson et al., 2013). Moreover, enriching a “suboptimal” 6.25 g dose of intact protein with additional leucine has been shown to increase the response of MPS equivalent to an “optimal” 25 g protein dose at rest (Churchward-Venne et al., 2012). However, studies investigating, the influence of leucine co-ingestion with EAA (Glynn et al., 2010), or intact protein (Koopman et al., 2005, 2006, 2008; Tipton et al., 2009; Wall et al., 2013) on the stimulation of MPS in young men report equivocal results. Whereas, the addition of leucine to whey protein failed to enhance the post-exercise response of MPS (Koopman et al., 2005, 2008; Tipton et al., 2009), adding leucine to a casein protein source was shown to increase the resting postprandial stimulation of MPS (Wall et al., 2013). While the response of MPS to leucine provision, with or without an intact protein source, is well-characterized, to our knowledge no study has successfully determined the response of MPS to the independent ingestion all three BCAAs (leucine, isoleucine, and valine) combined (i.e., without concurrent ingestion of intact protein or other macronutrients) following resistance exercise.

There is ample evidence that BCAA ingestion stimulates mTORC1 signaling (Karlsson et al., 2004; Apro and Blomstrand, 2010; Moberg et al., 2016), however the post-exercise response of MPS to BCAA ingestion remains unknown. A recent investigation with a similar design reported MPS data, but the very large variability in generated fractional synthetic rate (FSR) values precluded a solid conclusion from being drawn. Therefore, given uncertainty due to the known disconnect between signaling and MPS (Witard et al., 2009; Atherton et al., 2010a) and previous methodological difficulties in other research groups (Moberg et al., 2016), the primary aim of the present study was to determine the response of myofibrillar-MPS to ingestion of a drink containing BCAAs, but no other EAA, protein, or macronutrients, following resistance exercise. In addition, we also measured the phosphorylation status (as a surrogate marker of activity) of proteins within the mTORC1 signaling pathway in response to BCAA ingestion. We hypothesized that BCAA ingestion would increase both the stimulation of myofibrillar-MPS and the phosphorylation status of mTORC1-S6K1 signaling proteins following resistance exercise.

## Materials and methods

### Participants and ethics approval

Eleven healthy (body mass: 83.3 ± 11.7 kg; percent lean mass: 86.1 ± 4.1%) young (20.1 ± 1.3 years) men who regularly participated in resistance training of the lower body (≥2/week for >1 years) volunteered to participate in the present study. *A priori*, we conducted a power calculation (GPower v3 software) of appropriate sample size based on our previous published data (Witard et al., 2014) that measured, on average, a 37% higher post-exercise response of myofibrillar-MPS to 20 g of ingested whey protein compared with 0 g using the same tracer technique. The BCAA trial in the present study contained a similar quantity of BCAAs as the 20 g bolus of whey protein administered in our previous study (Witard et al., 2014; 5.6 vs. 4.8 g). By setting statistical power (1-ß err prob) at 0.8, α error probability at 0.05 and effect size (Cohen's *d*) at 1.4 (based on our previous data; Witard et al., 2014), our power calculation revealed a minimum sample size of 10 participants (using a crossover research design) would be necessary to detect a statistical difference in myofibrillar-MPS between BCAA and placebo trials. Due to illness, only 10 participants completed both experimental trials, therefore all data are shown for 10 participants.

The design, purpose, and potential risks associated with the study were explained to participants before obtaining written informed consent. Exclusion criteria were metabolic disorders, food intolerances/allergies, allergies to local anesthetic, current participation in other clinical trials, blood donations within three months of the initial screening visit, and the prescription of medication or consumption of nutritional/dietary supplements suggested to affect protein metabolism. The National Research Ethics Service board (Warwickshire, Birmingham, UK) approved all procedures and all were in accordance with the Helsinki Declaration of 1975 as revised in 1983. This trial is registered as a clinical trial (registration number: ISRCTN98737111).

### Experimental design

Participants reported to the laboratory on five separate occasions, including two experimental trials that were separated by approximately three weeks. During the initial visit, body composition was assessed using dual-energy x-ray absorptiometry (DEXA; Hologic Discovery W, Hologic Inc., Bedford, Massachusetts, USA) and one repetition maximum (1RM) was predicted for each leg individually on both leg press and leg extension. Approximately one week later, participants returned to the laboratory to confirm their 1RM for each leg. Three days later, participants performed their first blinded trial in which they consumed either a BCAA containing drink (BCAA; GlaxoSmithKline, Brentford, UK) or placebo (PLA) drink (GlaxoSmithKline, Brentford, UK; Table 1). BCAA contained 5.6 g of BCAAs which is equivalent to the typical BCAA content of 20 g whey protein. Participants performed a unilateral bout of resistance exercise prior to consuming the test drink in each trial. Myofibrillar-MPS was determined by measuring the incorporation of L-[ring-^13^C_6_] phenylalanine into myofibrillar protein during a primed continuous infusion. The complete testing procedure was repeated on the contralateral leg approximately three weeks after the first trial. Trial and exercised leg order were counter-balanced and randomized.

**Table 1:** Nutritional composition of drinks.

	**PLA**	**BCAA**
Calories (Kcal)	13	13
Leucine (g)	0	2.6
Isoleucine (g)	0	1.4
Valine (g)	0	1.6
Carbohydrate (g)	4.6	3.1
Fat (g)	0.02	0.01
Sodium (g)	0.2	0.2

*Drinks were consumed after a unilateral bout of resistance exercise and were either a branched-chain amino acid containing drink (BCAA) or a placebo (PLA) drink. BCAA contained 5.6 g of branched-chain amino acids which is equivalent to the typical branched-chain amino acid content of 20 g whey protein*.

### Preliminary testing

#### Exercise sessions

At least six days before and <2 week prior to the first infusion trial, 1RM was predicted for both legs of each participant on the leg press and leg extension using a previously published method (Verdijk et al., 2009). Briefly, volunteers warmed up each leg before completing the maximum number of repetitions at 80% of predicted 1RM. This exercise load was then inserted into an equation as previously reported to estimate 1RM (Mayhew et al., 1995).

Three days prior to both infusion trials, participants returned to the laboratory to confirm their 1RM on both exercises (Kraemer and Fry, 1995) on the leg that was to be exercised in the forthcoming trial. Briefly, the load was set to 90% of estimated 1RM from the previous session. Participants completed the maximum number of repetitions possible. The load was then increased by 5–10% until only one repetition could be completed. A rest period of 3 min was given between each load.

### Body composition, dietary control, and physical activity

During the initial visit, body composition (total and segmental) was assessed using DEXA. Prior the experimental trial, participants completed a questionnaire of food preferences and a 3-day diet diary that represented their habitual daily intakes. The average energy intake (3,392 ± 1,069 kcal) and macronutrient composition [protein: 2.2 ± 1.0 g·kgBM^−1^·d^−1^; carbohydrate: 4.7 ± 1.6 g·kgBM^−1^ ·d^−1^; fat: 1.4 ± 0.4 g·kgBM^−1^·d^−1^] from the 3 day diet diary was used to calculate the participant's diet before the experimental trial. Food parcels, which matched each participant's habitual energy and macronutrient intakes, were supplied for 48 h before the experimental trial. Participants were instructed to consume only food and drink sources provided by investigators and to consume their final meal no later than 22:00 on the evening before the experimental protocol. Analyses of the diet diary and food prescription were performed by using a commercially available software program (Wisp v3; Tinuviel software). All participants were instructed to refrain from physical exercise for 48 h before the experimental trial.

### Experimental protocol

**Figure 1:**
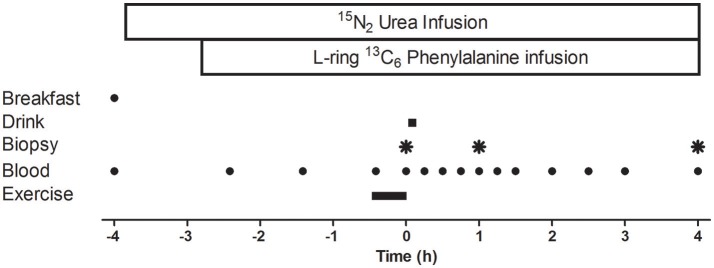
Schematic diagram of the infusion protocol. A baseline blood sample was collected before participants consumed an energy-rich, high-protein breakfast. A bout of unilateral leg-resistance exercise was performed 3 h after breakfast. Muscle biopsies (*vastus lateralis*) were collected from the exercised leg immediately prior (0 h), 1-, or 4 h-post drink ingestion. Drink ingestion was either a branched-chain amino acid containing beverage (BCAA) or placebo (PLA). Multiple blood samples were collected throughout the protocol. Ex, exercise.

A schematic diagram of the experimental protocol is displayed in Figure 1. Participants reported to the laboratory at ~06:15 following an overnight fast and measurements of height and weight were collected. A cannula was inserted into a forearm vein of one arm, and a resting blood sample was collected. Thereafter, participants were fed a standardized energy-rich (7 ± 1 kcal·kgBM^−1^), high protein (30 ± 1% of energy from protein, 50 ± 1% of energy from carbohydrate, and 20 ± 1% of energy from fat) breakfast. After breakfast a primed, continuous infusion (prime: ~88 μmol·kg^−1^; infusion rate: ~0.227 μmol·kgBM^−1^·min^−1^) of ^15^N_2_ urea was started and continued throughout the protocol. Subjects rested for ~75 min before a primed, continuous (2.0 μmol·kg^−1^; infusion ~0.05 μmol·kgBM^−1^.min^−1^) infusion of L-ring ^13^C_6_ phenylalanine (both Cambridge Isotope Laboratories Inc., Andover, Massachusetts, USA) was started.

Approximately 105 min later, participants performed a single bout of unilateral leg resistance exercise which lasted ~25 min. Participants completed a warm up on the leg press machine (Cybex VR3, Cybex International, Medway, Massachusetts, USA), which consisted of 12 repetitions at 40% 1RM, 10 repetitions at 50% 1RM, eight repetitions at 60% 1RM, and two repetitions at 70% 1RM. The warm up was followed by a 2 min rest period. The resistance exercise protocol consisted of four sets of 10 repetitions at 70% 1RM on the leg press machine and four sets of 10 repetitions at 75% 1RM on the leg extension machine (Cybex VR3, Cybex International, Medway, Massachusetts, USA). Participants rested for 2 min between each set and were free to consume water *ad libitum*. If a participant could not complete a full set, the load was lowered by 4.5 kg. Rating of perceived exertion (RPE) using the modified Borg scale (Borg, 1973) was measured after each set. Immediately (~5 min) after exercise (*t* = 0 min), an arterialized blood sample and a muscle biopsy were collected followed by ingestion of the test drink. Blood samples also were collected at multiple time points (*t* = −240, −145, −85, −25, 0, 15, 30, 45, 60, 75, 90, 120, 150, 180, 240 min) before and after drink ingestion. Two further muscle biopsies were collected 1 and 4 h after drink ingestion.

### Muscle biopsy collection and analysis

Muscle biopsies were obtained from the *vastus lateralis* of the exercised leg under local anesthesia (1% lidocaine) using 5 mm Bergstrom needles with suction. Different incisions (~1 cm apart, proximally from previous site) were used for each biopsy in an attempt to minimize the impact of local inflammation from the previous biopsy sample. Biopsy samples were immediately rinsed, blotted of excess blood, removed of visible fat and connective tissue, and divided into aliquots, before being frozen in liquid nitrogen, and stored at –80°C until later analysis. Muscle samples were analyzed for enrichment of L-[ring-^13^C_6_] phenylalanine in the intracellular pool and bound myofibrillar protein fractions, Furthermore, muscle was analyzed to measure the phosphorylation status of mTORC1-related signaling proteins.

### Myofibrillar protein enrichment

Muscle tissue was analyzed for enrichment of L-[ring-^13^C_6_] phenylalanine in the myofibrillar protein fraction. Myofibrillar proteins were isolated from ~30 mg tissue as previously described (Moore et al., 2009). Briefly muscle was snipped in ice-cold homogenizing buffer (50 mM Tris-HCL, 1 mM EDTA, 1 mM EGTA, 10 mM β-glycerophosphate, and 50 mM sodium fluoride). The homogenate was shaken for 10 min prior to being centrifuged at 1,000 g for 10 min at 4°C. The pellet was then re-suspended in homogenization buffer, before being shaken and centrifuged as described above. The myofibrillar fraction was separated from any collagen by dissolving the pellet in 0.3 M NaCl and heating for 30 min at 37°C. The proteins were then precipitated by combining the supernatant with 1 M PCA before being centrifuged for 20 min at 3500 rpm at 4°C. The pellet was then washed with 70% ethanol. The remaining myofibrillar pellet was hydrolyzed overnight at 110°C in 0.1 M HCl/Dowex 50W-X8 100–200 (Bio-Rad laboratories Inc., USA) and the liberated amino acids purified on cation-exchange columns (Bio-Rad laboratories Inc., USA). Amino acids were then converted to their N-acetyl-n-propyl ester derivative and phenylalanine labeling was determined by gas-chromatography-combustion-isotope ratio mass spectrometry (GC-C-IRMS, Delta-plus XL, Thermofinnigan, Hemel Hempstead, UK). Ions 44/45 were monitored for unlabeled and labeled CO_2_, respectively.

### Intracellular phenylalanine enrichment

Intracellular amino acids were liberated from ~20 mg of muscle. The frozen tissue was powdered under liquid nitrogen using a mortar and pestle and 500 μL of 1 M perchloric acid (PCA) was added. The mixture was centrifuged at 10,000 g for 10 min. The supernatant was then neutralized with 2 M potassium hydroxide and 0.2 M PCA and combined with 20 μL of urease for removal of urea. The free amino acids from the intracellular pool were purified on cation-exchange columns as described above. Intracellular amino acids were converted to their MTBSTFA derivative and ^13^C_6_ phenylalanine enrichment was determined by monitoring at ions 234/240 using gas chromatography mass spectrometry (GCMS; model 5973; Hewlett Packard, Palo Alto, CA).

### Western blotting

Muscle tissue (20–30 mg) was powdered on dry ice and then homogenized in lysis buffer (50 mM Tris pH 7.5; 250 mM Sucrose; 1 mM EDTA; 1 mM EGTA; 1% Triton X-100; 1 mM NaVO_4_; 50 mM NaF; 0.50% PIC) using a hand-held homogenizer (PRO200, UK). Samples were placed on a shaker for 1 h at 4°C, before being centrifuged for 5 min at 6,000 g. The supernatant was then used for determination of protein. A DC protein assay (Bio Rad, Hertfordshire, UK) was used for determining protein concentration. Equal amounts of protein were then boiled in Laemmli sample buffer (250 mM Tris-HCl, pH 6.8; 2% SDS; 10% glycerol; 0.01% bromophenol blue; 5% β-mercaptoethanol) and separated on SDS polyacrylamide gels (10–15%) for ~1 h at 58 mA. Proteins were then transferred to a Protran nitrocellulose membrane (Whatman, Dassel, Germany) at 100 V for 1 h. Membranes were blocked using milk solution and then incubated overnight at 4°C with the appropriate primary antibody. The primary antibodies used were: S6K1^Thr389^ (Cell signaling #9234); Akt^Ser473^ (Cell signaling #3787); 4E-BP1^Thr37/45^ (Santa Cruz SC6025); and PRAS40^Thr246^ (Cell Signaling #2610). The following morning the membrane was rinsed in wash buffer (TBS with 0.1% Tween-20) before being incubated for 1 h at room temperature with the appropriate secondary antibody; either horseradish (HRP)-linked anti-mouse IgG (New England Biolabs, 7072;1:1,000) or anti-rabbit IgG (New England Biolabs, 7074; 1:1,000) diluted in wash buffer. The membrane was then cleared of the antibody using wash buffer. Antibody binding was detected using enhanced chemiluminescence (Millipore, Billerica, MA). Imaging and band quantification were carried out using a Chemi Genius Bioimaging Gel Doc System (Syngene, Cambridge, UK). Blots were stripped in Restore™ Western Blot Stripping Buffer (Pierce, Rockford, IL) for 20 min and, re-blocked in milk solution and the respective total protein antibodies [S6K1 (Santa Cruz #sc-230); Akt (Cell signaling #9272); 4E-BP1 (Santa Cruz #sc-4251); and PRAS40 (Cell Signaling #2691)] were used to assure equal loading.

### Blood collection and analysis

Blood was collected in lithium heparin, EDTA-containing and serum separator tubes and centrifuged at 3,500 rpm for 15 min at 4°C. Aliquots of plasma and serum were then frozen at −80°C until later analysis. Plasma glucose and urea concentrations were analyzed using an instrumentation laboratory automated blood metabolite analyzer (Instrumentation Laboratory 650, Instrumentation Laboratory, Cheshire, UK). Serum insulin concentrations were measured using a commercially available ELISA (DRG Diagnostics, USA) following manufacturer's instructions.

### Plasma amino acid concentrations and enrichments

The ^13^C_6_ enrichments of phenylalanine and tyrosine were determined by GCMS by monitoring ions 234 and 240 for unlabeled and labeled phenylalanine and ions 466 and 472 for unlabeled and labeled tyrosine. Once thawed, plasma samples were mixed with diluted acetic acid and purified using a cation-exchange column (Bio-Rad laboratories Inc., USA). The amino acids were then converted to their N-tert-butyldimethyl-silyl-N-methyltrifluoracetamide (TBDMS) derivative.

Simultaneously, concentrations of phenylalanine, leucine, threonine, isoleucine, and valine were determined using an internal standard method (Tipton et al., 1999b, 2001). The selected amino acids were chosen to monitor blood concentrations of essential, non-essential, and the BCAAs. Briefly, plasma samples were weighed to obtain ~300 μL, 30 μL of an internal standard containing U-^13^C_9_-^15^N phenylalanine (ions 336/346), U-^13^C_6_ leucine, and isoleucine (ions 302/308), U-^13^C_4_-^15^N threonine (ions 405/409), and U-^13^C_5_ valine (ions 288/293) was then added and weighed. Since the weight of both sample and internal standard was known, it was possible to calculate a tracer-to-tracee ratio. Since the amino acid concentrations of the internal standard were known, it was possible to convert the tracer-to-tracee ratio into a concentration of each amino acid in plasma.

### Plasma urea enrichments

To determine ^15^N_2_ urea enrichments, 10 μL of plasma was mixed with 120 μL ethanol. Samples were then left in the fridge for 30 min prior to centrifugation at 13,000 rpm for 20 min at 4°C. The supernatant was then removed and dried under nitrogen. TBDMS and acetonitrile were added to the dried sample prior to heating at 90°C for 90 min. Samples were then run on GCMS and ions 231 and 233 were monitored.

### Calculations

#### Myofibrillar MPS

The fractional synthesis rate (FSR) of the myofibrillar protein fraction was calculated over the 4 h period using the standard precursor-product equation below:

(1) $$\begin{array}{l} FSR\left(\text{\%}\cdot h^{-1}\right)=\left(\frac{E_{B3}-E_{B1}}{E_{IC}\cdot t}\right)\cdot 100 \end{array}$$

Where *E*_*B*_ (_*B*3_ is the bound ^13^C_6_ phenylalanine enrichment measured in the biopsy collected at the 4h time point, *B*_1_ is the bound ^13^C_6_ phenylalanine enrichment measured in the biopsy collected at the 0 h time point), *E*_*IC*_ is the average *IC* phenylalanine enrichment of biopsies collected at 0 and 4 h, and *t* is time of tracer incorporation (h).

#### Endogenous urea production rates

The rate of urea production over time (μmol·h^−1^·kgBM^−1^) was calculated as previously described (Equation 2; Jahoor and Wolfe, 1987).

(2) $$\begin{array}{l} Production\;=\;\left(\left(\frac{E_{ui}}{E_{up}}\right)-1\right)\cdot i \end{array}$$

where *i* is the infusion rate of the urea tracer (μmol·h^−1^·kgBM^−1^) and *E*_*up*_ and *E*_*ui*_ are the enrichments of urea in the plasma and infusate, respectively.

#### Phenylalanine kinetics

Whole body phenylalanine rate of appearance was calculated by dividing the infusion rate by plasma phenylalanine enrichment, as described previously (Tipton et al., 1996). Phenylalanine oxidation was calculated by using the phenylalanine balance model (Thompson et al., 1989). Briefly, whole body phenylalanine oxidation was determined from the calculation of the hydroxylation of L-[ring-^13^C_6_]phenylalanine to L-[ring-^13^C_6_]tyrosine (Equation 3; Thompson et al., 1989).

(3) $$\begin{array}{l} Qpt\;=\;\frac{P_{t}}{P_{p}}\cdot \frac{Q_{p}^{2}}{\left(\frac{E_{p}}{E_{t}}-1\right)\cdot \left(i_{p}+Q_{p}\right)} \end{array}$$

Where, *P*_*t*_/*P*_*p*_ is estimated to be 0.73 (Thompson et al., 1989). *Q*_*p*_ is the phenylalanine flux (μmol·h^−1^·kgBM^−1^), *i* is the infusion rate of the tracer (μmol·h^−1^·kgBM^−1^) and *E*_*p*_ and *E*_*t*_ are the respective enrichments of phenylalanine and tyrosine.

### Statistical analyses and data presentation

Total area under the curve (tAUC) for serum insulin concentrations, phenylalanine Ra, and phenylalanine oxidation rates were calculated using Graphpad Prism V5.0 (Graphpad software incorporation, La Jolle, California, USA). tAUC-values were calculated from drink ingestion to final blood sample and a baseline y-axis value of zero for each was used.

A two-way repeated measures ANOVA was used to analyse blood glucose, insulin, amino acid, and urea concentrations, whole body urea production rates, phenylalanine Ra, phenylalanine oxidation rates, and phenylalanine/tyrosine enrichments (within-subject factor: time point; between-subject factor: trial). Exercise variables, myofibrillar-MPS, and tAUC of serum insulin concentrations, and phenylalanine kinetics for the post-exercise period were analyzed using a paired samples one-tailed *t*-test. Western blot data were expressed as percentage change from *t* = 0 (immediately post-exercise muscle biopsy) and data were analyzed using a two-way repeated measures ANOVA. Given the large variability associated with phosphorylation measurements of anabolic cell signaling, *a priori* we decided to explore differences over time for each trial separately. Significance for all analyses was set at *P* < 0.05. Where significance was detected, a LSD correction was used in *post-hoc* analysis. All statistical tests were completed using statistical package for social sciences version 23.0 (IBM, Chicago, Illinois, USA). All values are presented as means ± SEM, unless otherwise stated.

## Results

### Exercise variables

The lean tissue mass of the exercised leg, measured prior to each experimental protocol, was similar between trials (*P* > 0.05). The total weight lifted throughout the exercise protocol (including warm-up) was similar between BCAA (11,055 ± 837 kg) and PLA (11,089 ± 913 kg) trials (*P* = 0.88) and participants reported RPE scores 19 in all exercise trials.

### Blood glucose, insulin, and amino acid concentrations

Plasma glucose concentrations did not change over time (*P* = 0.093), were similar between trials (*P* = 0.365; data not shown) and no interaction effect was observed (*P* = 0.548). In both trials, serum insulin concentrations markedly (264%) increased (*P* < 0.01) in response to breakfast, before returning to baseline 1 h after exercise and remained stable over the remainder of the recovery period (data not shown). There were no differences in insulin concentrations between BCAA and PLA trials (*P* = 0.48) and no interaction effect was observed (*P* = 0.36). The insulin tAUC over 4 h following drink ingestion was similar (*P* = 0.70) between BCAA (50 ± 17 μIU·mL^−1^^*^240 min) and PLA (53 ± 19 μIU·mL^−1^^*^240 min) trials.

**Figure 2:**
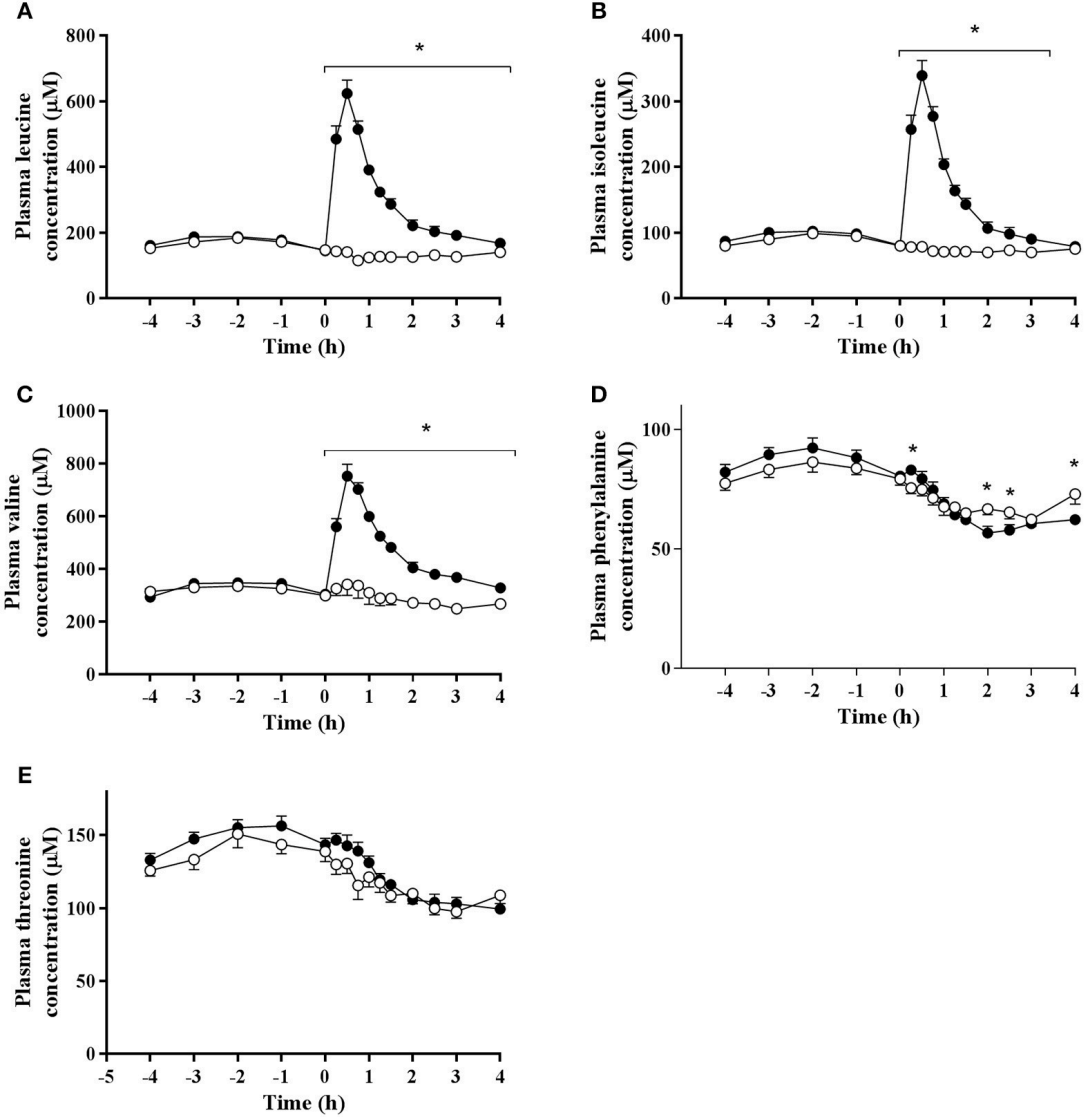
Plasma concentrations of **(A)** leucine, **(B)** isoleucine, **(C)** valine, **(D)** phenylalanine, and **(E)** threonine pre and post ingestion of either a branched-chain amino acid containing drink (BCAA, closed circles) or placebo drink (PLA, open circles) following resistance exercise. Data are displayed as means ± SE. ^*^Significant difference between trials (*P* < 0.05).

A significant time effect was observed for plasma concentrations of leucine (*P* < 0.001), isoleucine (*P* < 0.001), and valine (*P* < 0.001; Figure 2). The concentrations of all branched chain amino acids were greater in BCAA than PLA (*P* < 0.001). Peak leucine (624 ± 41 μM), isoleucine (339 ± 23 μM), and valine (753 ± 45 μM) concentrations were observed at 0.5 h-post drink ingestion in BCAA.percentage increase from baseline of 300 ± 96% (leucine), 300 ± 88% (isoleucine), and 144 ± 59% (valine). A significant interaction effect for plasma branched chain amino acid concentrations was observed whereby over the entire measurement period after drink ingestion, leucine (*P* < 0.001), and valine (*P* ≤ 0.002) concentrations were greater in BCAA than PLA. Over a 3.5 h period post drink ingestion, isoleucine levels were higher in BCAA (*P* ≤ 0.001).

Phenylalanine and threonine concentrations declined to below baseline values after drink ingestion in both trials (*P* < 0.001). There were no difference in threonine concentrations between trials (*P* = 0.207) and no interaction effect was observed (*P* = 0.056). Phenylalanine concentrations (main effect of trial; *P* = 0.003) were significantly higher in BCAA than PLA at 15 min (83 ± 5 vs. 75 ± 8 μM, *P* = 0.018), but significantly lower in BCAA than PLA at 120 min (57 ± 9 vs. 67 ± 8 μM, *P* = 0.003), 150 min (58 ± 7 vs. 65 ± 9 μM, *P* = 0.007), and 240 min (63 ± 6 vs. 73 ± 13 μM, *P* = 0.021) post drink ingestion.

### Blood urea concentrations and whole-body urea production rates

A decrease in plasma urea concentrations was observed in both trials during the latter stage (3–4 h) of exercise recovery (*P* < 0.026), with no differences between trials (*P* = 0.733; data not shown). Whole body urea production rates decreased from immediately post-exercise to the end of the 4 h recovery period (*P* < 0.001; data not shown), however there was no differences between trials (*P* = 0.685) and no interaction effect was observed (*P* = 0.611). The tAUC of urea production in response to post-exercise drink ingestion was similar between BCAA (2,314 ± 490 μmol·kgBM^−1^) and PLA (2,272 ± 636 μmol·kgBM^−1^ *P* = 0.694) trials.

### Amino acid enrichments

**Figure 3:**
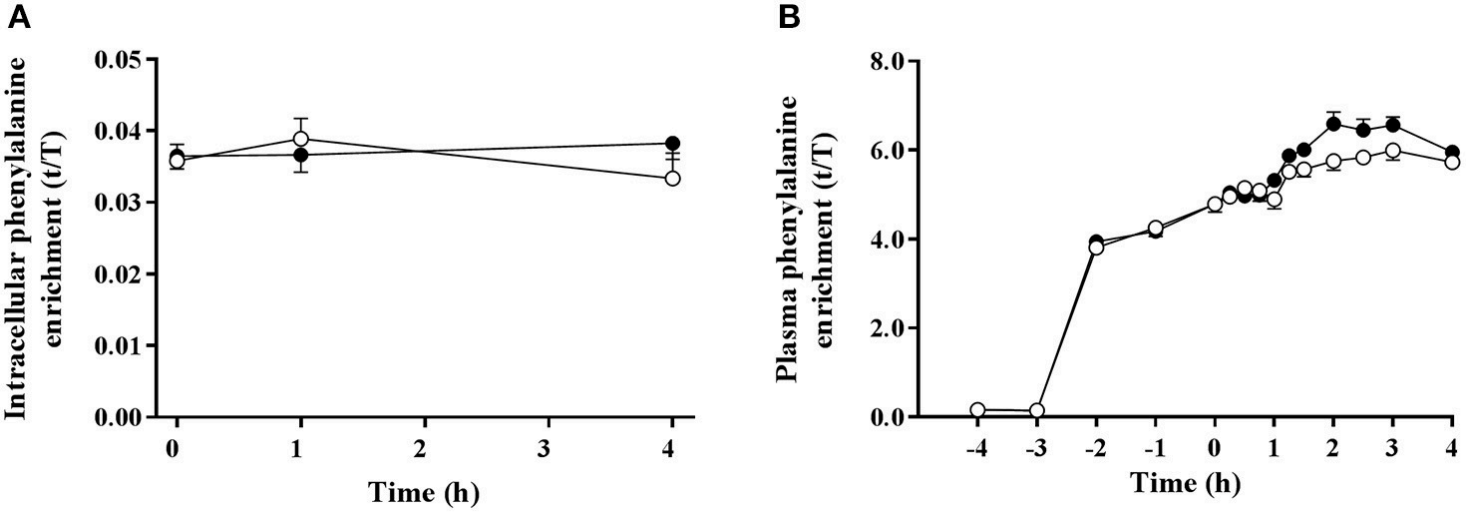
Muscle intracellular **(A)** and plasma **(B)** ^13^C_6_ phenylalanine enrichments pre and post ingestion of either a branched-chain amino acid containing drink (BCAA, black circles) or placebo drink (PLA, white circles) following resistance exercise. Data are displayed as means ± SE.

Muscle intracellular and blood plasma ^13^C_6_ phenylalanine enrichments were stable over the measured time period of tracer incorporation (0–4 h) in both trials (Figure 3). Plasma ^13^C_6_ tyrosine enrichments increased over time during the experimental protocol (data not shown). Trial order had no effect on intracellular and plasma tracer enrichments.

### Whole body phenylalanine kinetics

**Figure 4:**
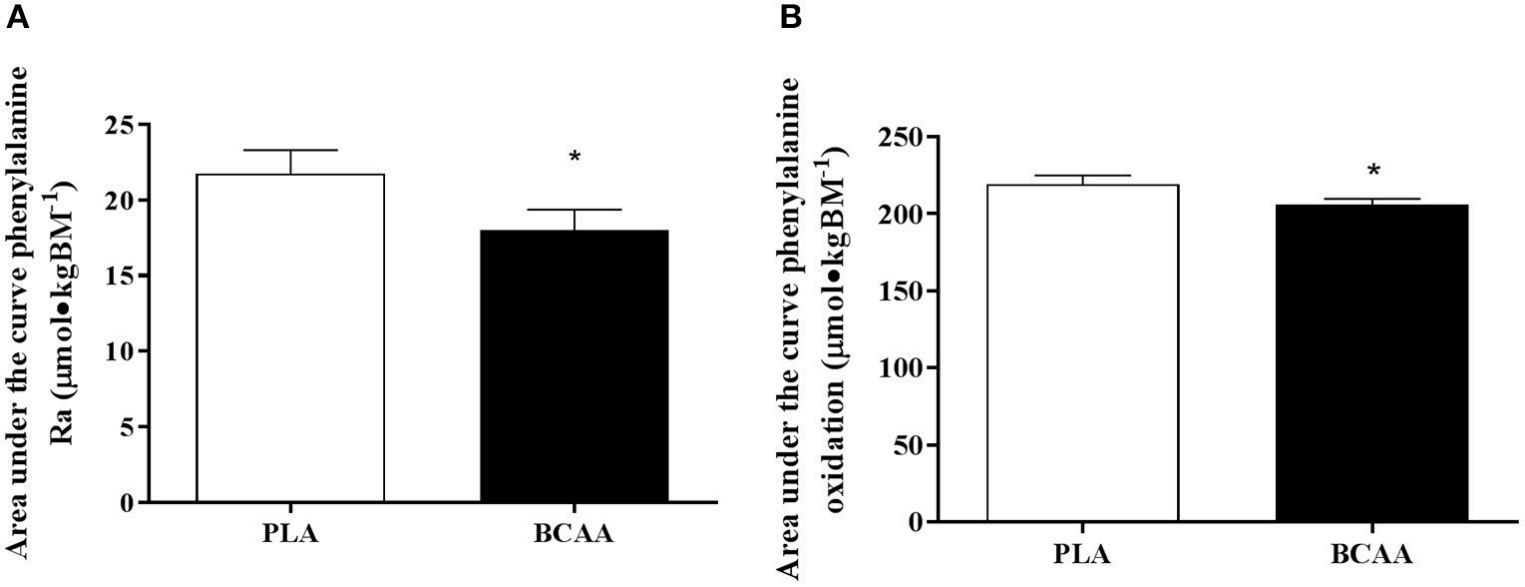
Phenylalanine kinetics, expressed as, total area under the curve for phenylalanine rate of appearance **(A)** and total area under the curve for phenylalanine oxidation **(B)** following the post-exercise ingestion of a branched-chain amino acid (BCAA, black bars) or placebo drink (PLA, white bars). Data are displayed as means ± SE. ^*^Significant difference compared with PLA (*P* < 0.05).

Phenylalanine Ra, expressed as tAUC over the post drink period, was 6% lower in BCAA than PLA (*P* = 0.028). In addition, tAUC of whole body phenylalanine oxidation rate was 16% lower in BCAA compared with PLA (*P* = 0.006, Figure 4).

### Anabolic signaling proteins

**Figure 5:**
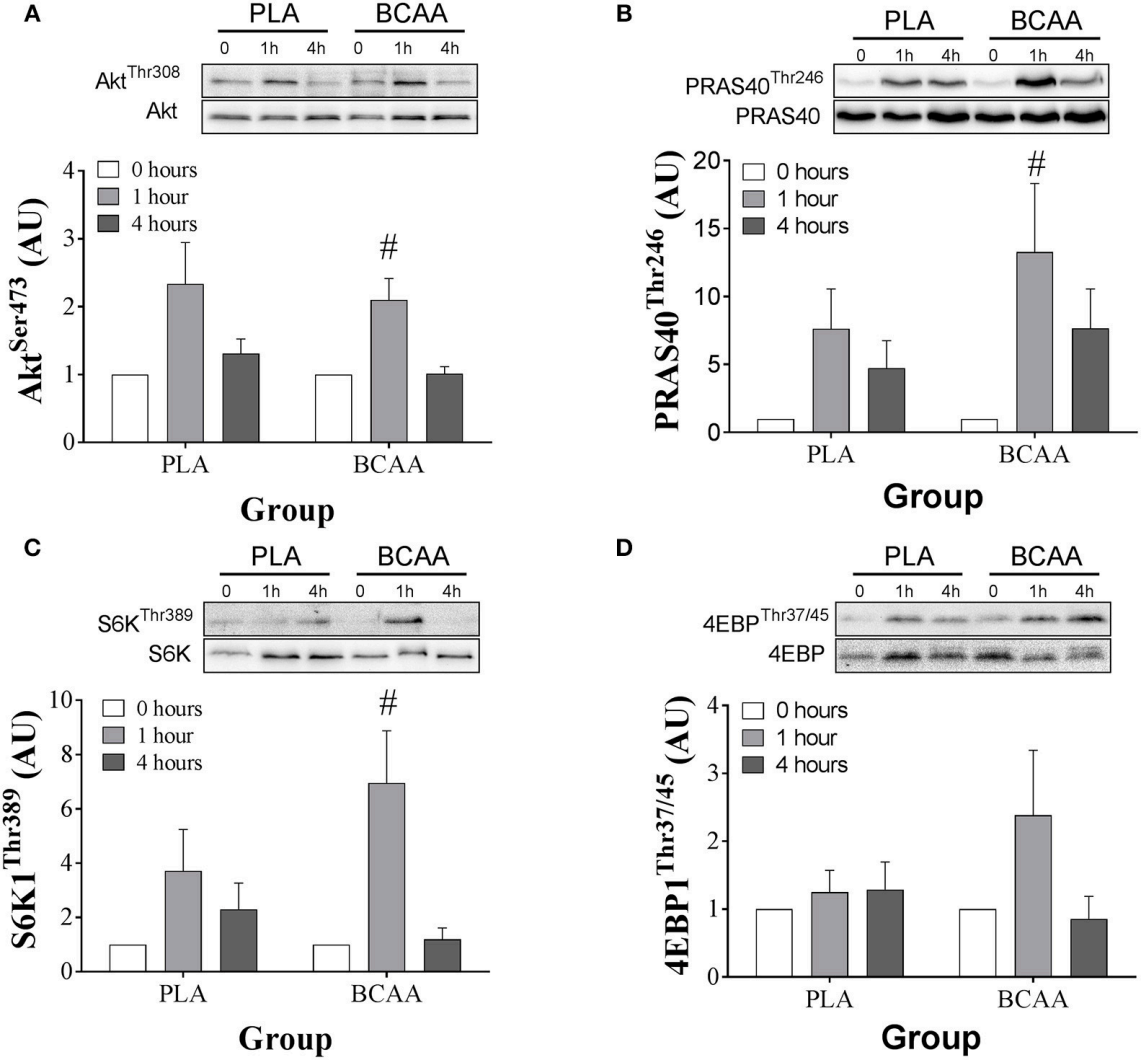
Phosphorylation status of, AKT^Ser473^ **(A)**, PRAS40^Thr246^ **(B)**, S6K1^Thr389^ **(C)**, and 4EBP1^Thr37/45^ **(D)** immediately pre (0 h), 1-, and 4 h-post ingestion of either a branched-chain amino acid containing drink (BCAA, black bars) or placebo drink (PLA, white bars) following resistance exercise. Data are displayed as means ± SE. ^#^Significant difference from 0 h in respective trials (*P* < 0.05).

Signaling data are displayed in Figure 5. The phosphorylation of AKT^Ser473^ increased over time (*P* = 0.025). However, there was no trial effect (*P* = 0.388). The phosphorylation of AKT^Ser473^ increased at 1 h compared with 0 h in BCAA (*P* = 0.007). Further, phosphorylation of AKT^Ser473^ was higher at 1 h compared with 4 h in BCAA (*P* = 0.015) but there were no differences between 0 and 4 h in BCAA (*P* = 0.903). There were no differences between 0 and 1 h (*P* = 0.056) or 4 h (*P* = 0.174) and 1 and 4 h (*P* = 0.085) in PLA.

The phosphorylation of PRAS40^Thr246^ increased over time (*P* = 0.022). However, there was no trial effect (*P* = 0.281). The phosphorylation of PRAS40^Thr246^ increased ~12-fold at 1 h compared with 0 h in BCAA (*P* = 0.037). Further phosphorylation of PRAS40^Thr246^ was higher at 1 h compared with 4 h in BCAA (*P* = 0.047) but there were no differences between 0 and 4 h in BCAA (*P* = 0.102). There were no differences between 0 and 1 h (*P* = 0.050) or 4 h (*P* = 0.101) and 1 and 4 h (*P* = 0.112) in PLA.

The phosphorylation of S6K1^Thr389^ increased over time (*P* = 0.021). However, there was no trial effect (*P* = 0.087). The phosphorylation of S6K1^Thr389^ increased ~6-fold at 1 h compared with 0 h in BCAA (*P* = 0.017). Further, phosphorylation of S6K1^Thr389^ was higher at 1 h compared with 4 h in BCAA (*P* = 0.014) but there were no differences between 1 and 4 h in BCAA (*P* = 0.690). There were no differences between 0 and 1 h (*P* = 0.120) or 4 h (*P* = 0.05)0 and 1 and 4 h (*P* = 0.795) in PLA.

The phosphorylation of 4EBP1^Thr37/45^ remained constant over time (*P* = 0.115) and there were no trial effect (*P* = 0.566).

### Muscle myofibrillar protein synthesis

**Figure 6:**
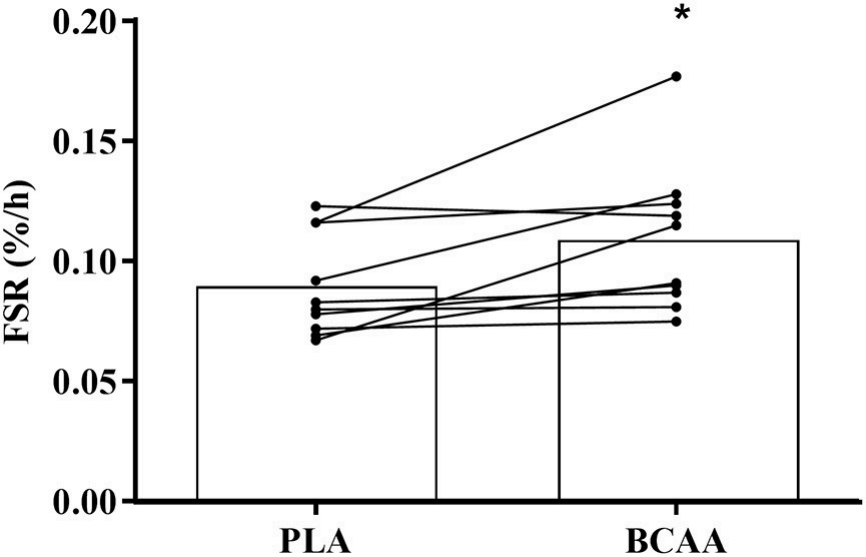
Muscle myofibrillar fractional synthesis rate following the post-exercise ingestion of a branched-chain amino acid containing drink (BCAA) or placebo drink (PLA). Data are displayed as means (bars) and individual responses (dots and lines). ^*^Significant difference compared with PLA (*P* < 0.05).

Myofibrillar FSR was ~22% higher (*P* = 0.012) in BCAA than PLA over the 4 h period following drink ingestion (Figure 6).

## Discussion

The present study demonstrated that ingesting of all three BCAAs alone, without concurrent ingestion of other EAA, protein, or macronutrients, stimulated a 22% greater response of myofibrillar-MPS following resistance exercise compared with a placebo. The magnitude of this increased response of myofibrillar-MPS was ~50% less than the previously reported myofibrillar-MPS response to a dose of whey protein containing similar amounts of BCAAs (Churchward-Venne et al., 2012; Witard et al., 2014). Taken together, these results demonstrate that BCAAs exhibit the capacity to stimulate myofibrillar-MPS, however a full complement of EAA could be necessary to stimulate a maximal response of myofibrillar-MPS following resistance exercise. This information potentially has important nutritional implications for selecting amino acid supplements to facilitate skeletal muscle hypertrophy in response to resistance exercise training and the maintenance of muscle mass during aging, unloading, or disease.

The most likely physiological explanation for the apparent attenuation of the post-exercise response of myofibrillar-MPS to BCAA ingestion in comparison to an intact protein source relates to the limited availability of amino acids as substrate for MPS. It is well-established that BCAA ingestion stimulates the activation of mTORC1 signaling pathways that regulate the translational activity of MPS (Karlsson et al., 2004; Apro and Blomstrand, 2010; Moberg et al., 2016). Moreover, recent results demonstrate that the presence of the valine and isoleucine enhances the response of mTORC1 to leucine (Moberg et al., 2016). However, results from the present study suggest that ingesting BCAAs alone, without the other EAA, provides limited substrate for protein synthesis in exercised muscles. Thus, the overall response of MPS is not maximized. Instead, the limited availability of EAA likely explains the qualitative difference in magnitude of the MPS response to ingestion of BCAAs alone and ingestion of similar amounts of BCAAs as part of intact whey protein (Churchward-Venne et al., 2012, 2014; Witard et al., 2014). Moreover, in the present study, we observed a decline in arterialized phenylalanine concentrations 3 h after drink ingestion in the BCAA trial. This finding is consistent with previous research that observed decreased EAA concentrations following leucine ingestion (Hagenfeldt and Wahren, 1980; Nair et al., 1992; Tipton et al., 2009; Borgenvik et al., 2012). Taken together, these data support the notion that EAA availability is the rate-limiting factor for stimulating a maximal MPS response to resistance exercise with BCAA ingestion.

The decline in EAA availability that we observed with BCAA ingestion also provides a potential physiological explanation for the differential response of MPS to ingesting a BCAA source at rest and following resistance exercise. Previously, Wilkinson and colleagues reported an ~100% increase in myofibrillar-MPS in response to ingestion of leucine alone at rest (Wilkinson et al., 2013). Furthermore, a study from Churchward-Venne and colleagues reported that adding leucine to a “suboptimal” dose (6.25 g) of whey protein resulted in rates of myofibrillar-MPS similar to those after ingestion of 25 g of whey protein at rest. However, consistent with the results reported in the present study, the addition of leucine to 6.25 g of whey protein was not as effective as higher doses of whey protein ingested after resistance exercise (Churchward-Venne et al., 2012). Thus, exercise has been shown to alter the relationship of the MPS response to ingested leucine and intact protein. It is likely that the enhanced ability of muscle to utilize ingested protein following exercise (Pennings et al., 2011; Witard et al., 2014) leads to a greater demand for EAA, thus resulting in limited EAA availability at the greater rates of MPS. In another study, Churchward-Venne et al. (2014) also reported that BCAA added to a suboptimal dose of whey protein resulted in less stimulation of MPS than when the same amount of leucine alone was added to suboptimal whey protein. These data suggest that leucine alone may be sufficient to stimulate MPS following exercise provided a minimal amount of EAA is included. However, Churchward-Venne et al. (2014) included ingestion of leucine with 6.25 g of whey protein and a mixed macronutrient beverage making direct comparisons with our results difficult. Leucine seems to be the most important BCAA for stimulation of the translation initiation pathways (Anthony et al., 2000; Crozier et al., 2005) and leucine alone stimulates MPS at rest (Wilkinson et al., 2013). Thus, it is possible that leucine is solely responsible for the stimulation in MPS we report here. On the other hand, recent data suggest that there is a greater stimulatory effect on mTORC1 when valine and isoleucine are consumed alongside leucine (Moberg et al., 2016). Nevertheless, taken together, these past and present data suggest that the availability of EAA may be a critical factor for the optimal response of MPS following resistance exercise. Since the ingestion of BCAAs alone stimulates myofibrillar-MPS, but does not increase the supply of all EAA, the overall response of myofibrillar-MPS following resistance exercise is limited.

The influence of exogenous BCAAs on the stimulation of MPS following exercise is mediated by an increased activation of the mTORC1-S6K1 signaling pathway. In the present study, the stimulation of PRAS40^Thr246^, and S6K1^Thr389^ phosphorylation 1 h following drink ingestion was higher in BCAA than PLA. Thus, our results are consistent with earlier work demonstrating an upregulation of translational activity with BCAA ingestion (Karlsson et al., 2004; Apro and Blomstrand, 2010). The present study is the first to demonstrate the upregulation of mTORC1 signaling with BCAA ingestion translates into an increased response of myofibrillar-MPS following resistance exercise.

BCAAs has been shown to influence muscle protein metabolism through MPB as well as MPS (Buse and Reid, 1975; Louard et al., 1990; Wilkinson et al., 2013). Early studies demonstrated that BCAAs reduce whole-body protein breakdown (Ferrando et al., 1995) and MPB (Louard et al., 1990; Nair et al., 1992). Recent evidence suggests the impact of BCAAs on MPB may be mediated by the leucine metabolite, β-hydroxy-β-methylbutyrate (Wilkinson et al., 2013). However, to date no study has investigated the impact of BCAA supplementation on MPB following exercise. Unfortunately, it was not possible to directly measure MPB in the present study, however our data suggest that whole-body protein breakdown was reduced by BCAA ingestion. Whereas, urea production rates were similar between trials, phenylalanine Ra, and phenylalanine oxidation rates—both indicators of whole body protein catabolism—were lower in BCAA than PLA during the 4 h period after drink ingestion. These data are consistent with previously reported results following ingestion of intact protein with similar amounts of BCAAs (Witard et al., 2014). It has been reported that MPB is reduced by insulin following resistance exercise (Biolo et al., 1999). However, the insulin response to BCAA and PLA was similar, so it is unlikely that insulin explains the decline in whole-body protein breakdown following BCAA ingestion. Thus, it seems clear that whole-body protein breakdown is decreased, albeit minimally, with ingestion of BCAAs following resistance exercise. However, these results do not necessarily mean that MPB is decreased with BCAA ingestion.

To conclude, the ingestion of BCAAs alone, without the concurrent ingestion of other EAA, intact protein or other macronutrients, increases the stimulation of mTORC1 activity and myofibrillar-MPS following exercise in resistance-trained young men. Our data support the notion that BCAA ingestion alone does not maximally stimulate myofibrillar-MPS following exercise despite stimulation of translation initiation pathways. The lack of sufficient EAA appears to limit the response of myofibrillar-MPS following exercise. Thus, whereas our data clearly show that BCAA ingestion activates cell-signaling pathways that result in increased myofibrillar-MPS, ingestion of BCAAs alone may not be the optimal nutritional regimen to stimulate a maximal MPS response to resistance exercise training.

## Author contributions

SRJ, OCW, GAW, and KDT designed the research project and conducted the research; SRJ, OCW, and KDT had primary responsibility for the final content of the manuscript and wrote the manuscript; SRJ, OCW, AP, and KB analyzed data or performed statistical analysis and all authors read and approved the final manuscript.

### Conflict of interest statement

The authors declare that the research was conducted in the absence of any commercial or financial relationships that could be construed as a potential conflict of interest.
